# The Bureau of Animal Industry

**Published:** 1885-04

**Authors:** 


### THE BUREAU OF ANIMAL INDUSTRY.

### ITS GREAT OPPORTUNITY.

With the change of Administration at Washington may come changes in this very important branch of our Government.

It is not going too far to say that the Bureau has not met the demands the country has a right to make upon it, even in things that are possible. Its quarantine arrangements are still insufficient, and fail in that exactness of minutiæ by which only a quarantine can be really said to be such. The genius to arrange these things properly has not yet been secured at Washington.

Another very important error has been in the manner of selecting the subordinates to the Chief Veterinarian. In the first place, the appointment of civilians in the Bureau, such as stock-men, is unwise, as these appointments are optional, not at all a necessity. In their stead two veterinary advisers should be appointed, to be called to Washington as consulting members, when necessary. We are bitterly opposed to appointing laymen on technical commissions. As Mr. Law has said, "they must first be instructed before they can know anything," and they never can be so instructed as to be anything but hindrances.

The manner of appointing the "Special Agents," as they are called, is clearly wrong. When Dr. Loring said they were equally as competent as the Chief Veterinarian of the Bureau, he either under-estimated the latter or greatly over-estimated the former, for not one of them knows sufficient pathology to occupy the position. There is but one way out of this difficulty, and if any one at Washington has any true purpose in him it will be adopted. That is, to establish a patho-mycological laboratory at Washington in connection with the Bureau, and to place at its head the ablest man they can get, capable of speaking and teaching the English language, and to have a sufficient number of assistants, and to give at least three months' instruction to these Special Agents before their definite appointment.

A still better way would be to continue the present men in office for six months, and to place the whole business open to public competition, the Government allowing every veterinarian who would come to such a laboratory to study, $100 a month to pay his expenses, and then to examine all candidates and select the best for its purposes. In founding such a laboratory the Agricultural Department would be taking the most needed step in an absolutely necessary direction, and it would undoubtedly lead to the formation of a National Veterinary, and in the end to a National Institute of Sciences, at Washington.

